# Insights Into the Regulation of Gynecological Inflammation-Mediated Malignancy by Metalloproteinases

**DOI:** 10.3389/fcell.2021.780510

**Published:** 2021-11-29

**Authors:** Yasmin Begum, Anuradha Pandit, Snehasikta Swarnakar

**Affiliations:** Infectious Diseases and Immunology Division, CSIR-Indian Institute of Chemical Biology, Kolkata, India

**Keywords:** inflammation, gynecological cancer, matrix metalloproteinase (MMP), A disintegrin and metalloproteinase (ADAM), A disintegrin and metalloproteinase with thrombospondin motif (ADAMTS), metastasis, angiogenesis

## Abstract

Gynecological illness accounts for around 4.5% of the global disease burden, which is higher than other key global health concerns such as malaria (1.04%), TB (1.9%), ischemic heart disease (2.2%), and maternal disorders (3.5%). Gynecological conditions in women of reproductive age are linked to both in terms of diagnosis and treatment, especially in low-income economies, which poses a serious social problem. A greater understanding of health promotion and illness management can help to prevent diseases in gynecology. Due to the lack of established biomarkers, the identification of gynecological diseases, including malignancies, has proven to be challenging in most situations, and histological exams remain the gold standard. Metalloproteinases (MMPs, ADAMs, ADAMTSs) and their endogenous inhibitors (TIMPs) modulate the protease-dependent bioavailability of local niche components (e.g., growth factors), matrix turnover, and cellular interactions to govern specific physical and biochemical characteristics of the environment. Matrix metalloproteinases (MMPs), A Disintegrin and Metalloproteinase (ADAM), and A Disintegrin and Metalloproteinase with Thrombospondin Motif (ADAMTS) are zinc-dependent endopeptidases that contribute significantly to the disintegration of extracellular matrix proteins and shedding of membrane-bound receptor molecules in several diseases, including arthritis. MMPs are noteworthy genes associated with cancer development, functional angiogenesis, invasion, metastasis, and immune surveillance evasion. These genes are often elevated in cancer and multiple benign gynecological disorders like endometriosis, according to research. Migration through the extracellular matrix, which involves proteolytic activity, is an essential step in tumor cell extravasation and metastasis. However, none of the MMPs’ expression patterns, as well as their diagnostic and prognostic potential, have been studied in a pan-cancer context. The latter plays a very important role in cell signaling and might be used as a cancer treatment target. ADAMs are implicated in tumor cell proliferation, angiogenesis, and metastasis. This review will focus on the contribution of the aforementioned metalloproteinases in regulating gynecological disorders and their subsequent manipulation for therapeutic intervention.

## Introduction

Gynecological malignancies, mainly comprised of cervical, ovarian, and endometrial cancers, contribute immensely to the worldwide cancer load, with cervical cancer being the fourth most common cancer in women (Bray et al., 2018). The high mortality rate of gynecological cancers is majorly due to late diagnosis of the disease, chemoresistance, impaired apoptotic pathway, persistent inflammation, and aberrant MMP production (Azevedo Martins et al., 2020; Carey et al., 2021; Chen et al., 2021). Inflammation is a process by which the innate immune system reacts to tissue injury or infection by bacteria, viruses, toxins, etc., and facilitates the recruitment of circulating cells by the adaptive immune system to the damaged tissue. In contrast to a normal inflammatory response, which is characterized by a temporally limited increase in inflammatory signals that resolves once the threat has passed, systemic chronic inflammation (SCI) is a low-grade, prolonged state of the activated immune response leading to changes in normal cellular morphology. SCI has been linked to increased risk of hypertension, hyperglycemia, diabetes, cardiovascular disease, chronic renal disease, osteoporosis, and different kinds of cancer (Golia et al., 2014; Chang and Yang, 2016; Suarez-Carmona et al., 2017; Cobo et al., 2018; Kanbay et al., 2018; Boni-Schnetzler and Meier, 2019). Invading pathogens release foreign peptides, carbohydrates, and nucleic acids that make up damage-associated molecular patterns (DAMPs) and recruit activated immune cells to the inflammation site. These components along with debris from tissue damage and extracellular matrix can bind to pattern recognition receptors (e.g., Toll-like receptors) on the surface of tissue-resident macrophages, dendritic cells, histiocytes, mast cells, and other granulocytes. Once activated, granulocytes secrete antimicrobial agents, enzymes, cytokines, reactive oxygen/nitrogen species (RONS), prostaglandins, leading to changes in blood vasculature, thereby enhancing the transport of circulating immune cells to the damage site and also sensitizing receptors to pain. (Coussens and Werb, 2002).

The relation between inflammation and cancer has long been established since the evidence showed that inflammation led to genetic instability and impaired DNA repair pathways. Tumors generate and maintain a local inflammatory response that allows cells to spread. Nuclear factor-kappa B (NF-κB), RONS, cytokines, prostaglandins, and specific microRNAs are all upregulated in the inflammatory milieu, affecting cell proliferation, cell death, cellular senescence, DNA damage, and angiogenesis (Baldwin, 2001; Munn, 2017; Srinivas et al., 2019; Ye et al., 2020). Cytokines like Interleukin-6 (IL-6), tumor necrosis factor-α (TNF-α), which are key players during an inflammatory response, have been linked to tumorigenesis and metastasis by influencing NF-κB and STAT3 signaling pathways (Liu et al., 2020a; Jin, 2020; Sreenivasan et al., 2020). TNF-α is known to enhance stemness *via* NF-κB in mammary cancer growth (Liu et al., 2020a).

Although inflammation is not the cause of all malignancies, the tumor microenvironment generates an inflammatory milieu that aids tumor development. Infiltrating immune cells and inflammatory mediators like cytokines/chemokines and growth factors contribute to cell proliferation and migration in multiple tumors by upregulating metalloproteinases. For solid tumor cells to disseminate to remote sites, extracellular matrix (ECM) remodeling *via* proteolytic cleavage is carried out by a category of enzymes called metalloproteinases. These majorly comprise of MMPs and ADAM/ADAMTS. Matrix metalloproteinases (MMPs) are a class of zinc-dependent endopeptidase that degrades most ECM components like collagen. MMPs along with their inhibitors TIMPs (Tissue Inhibitors of Metalloproteinases) are responsible for tissue turnover, wound healing, and morphogenesis (Patel et al., 2016; Chan et al., 2020). A Disintegrin and metalloproteinase (ADAM), and A Disintegrin and Metalloproteinase with Thrombospondin Motif (ADAMTS) are zinc-requiring proteases that degrade extracellular matrix proteins and shed membrane-bound receptor molecules in several diseases (Rowan et al., 2008; Jones et al., 2016; Malemud, 2019).

Increasing knowledge of the genetic pathways involved in cancer has resulted in the formation of several anticancer drugs. Despite years of dedicated cancer research, little is known about the role of inflammation in the advancement of cancer. Understanding the processes involved in cancer-related inflammation might lead to the development of synergistic medicines that target the inflammatory mediators and their downstream effectors modulating the tumor microenvironment.

### Gynecological Inflammation Associated Cancer Risk

Around 15% of all malignancies are caused due to chronic or persistent inflammation (Kuper et al., 2000). Persistent inflammation can perpetuate tumor progression and in turn, tumor-induced inflammation promotes cancer growth. Developing tumors disrupt normal tissue, producing DAMPs that activate receptors on local granulocytes, triggering inflammatory processes. Tumors compress the blood and lymphatic arteries, detaching oxygen and nutrition delivery, leading to hypoxia, which signals the production of cytokines and angiogenic growth factors, recruiting macrophages and immune cells to the inflammation site (Munn, 2017).

**FIGURE1:**
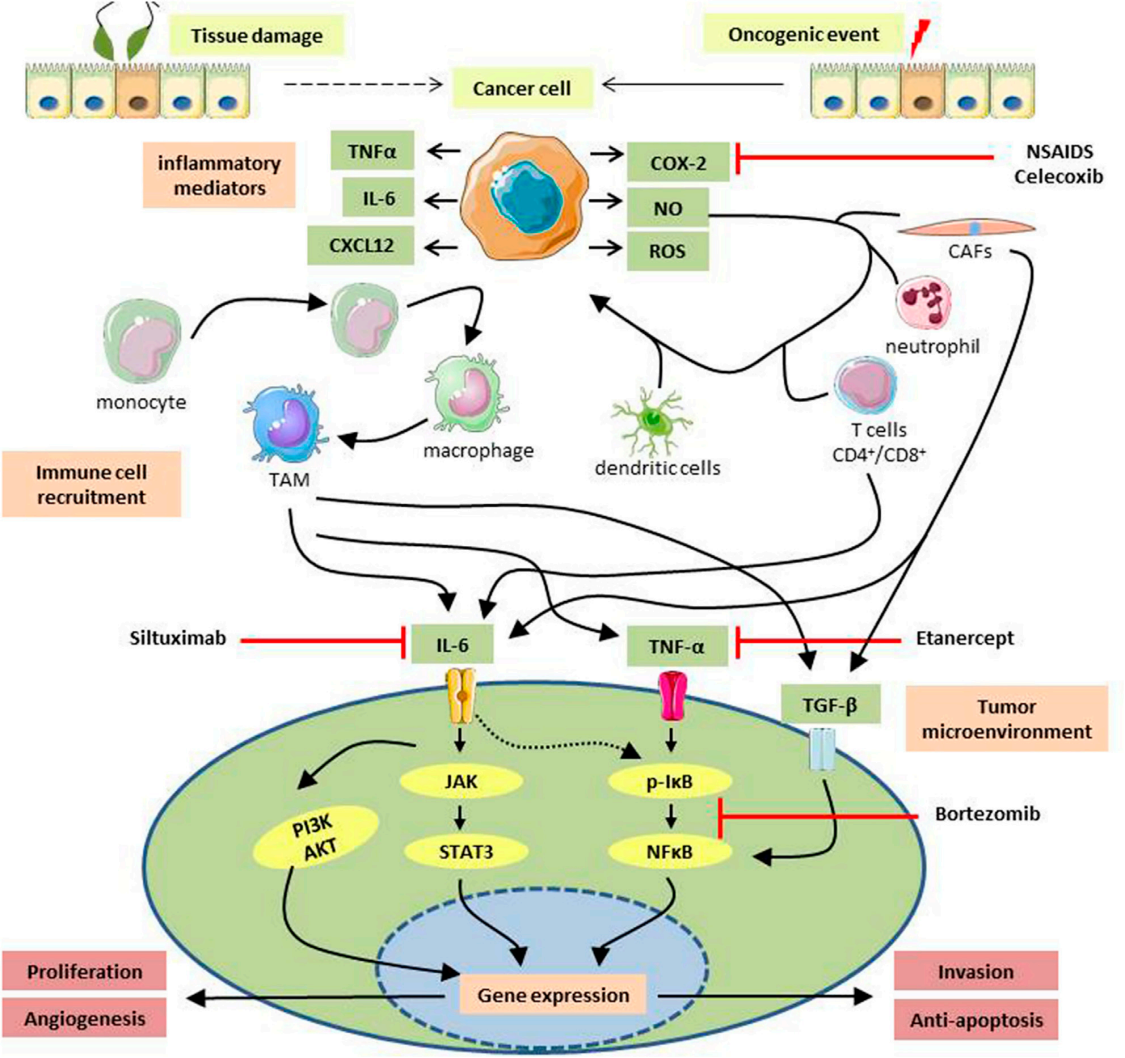
Diagrammatic representation of inflammation-induced tumorigenesis. Cellular damage caused by external agents, followed by subsequent inflammation or initiation of an oncogenic event might transform a healthy cell into a cancerous one. Cancer cells secrete a plethora of soluble factors like chemokines, interleukins, tumor necrotic factors, reactive oxygen/nitrogen species that attracts circulating immune cells to the vicinity. Infiltrated immune cells, e.g., monocytes, dendritic cells, cancer-associated fibroblasts, neutrophils, CD4^+^/CD8^+^ T cells, interact with the surrounding cancer cells and secrete potent mediators to establish an inflammatory milieu at the tumor site. A low-grade persistent inflammation formed as a result can activate various oncogenic signaling pathways which express oncogenes responsible for tumor growth, metastasis, neoangiogenesis, etc., and help in the progression of malignancies. Anti-inflammatory drugs used to treat gynecological cancers are NSAIDs e.g., celecoxib, COX-2 inhibitor; Etanercept, soluble TNF- α blocker; siltuximab, IL-6 blocking antibody; Bortezomib, an inhibitor of NFκB activation. TNF-α, Tumor-Necrotic Factor α; IL-6, Interleukin-6; CXCL12, CXC Motif Chemokine Ligand 12; COX-2, cyclooxygenase two; NO, Nitric Oxide; ROS, Reactive Oxygen Species; TAM, Tumor-Associated Macrophage; CAFs, Cancer-Associated Fibroblasts; TGF-β, Transforming Growth Factor β; JAK, Janus Activated kinase; STAT3, Signal Transducer And Activator Of Transcription three; PI3K, Phosphoinositide three kinase; AKT, Protein kinase B; IκB, Inhibitor Kappa B; NFκB, Nuclear Factor Kappa B.

Tumor cells secrete a variety of inflammatory mediators and cytokines/chemokines to recruit circulating immune cells to the tumor site. After infiltrating the tumor, myeloid, lymphoid, and mesenchymal cells activate various autocrine/paracrine signaling pathways and amplify the inflammatory reaction. Infiltrating cells can produce a plethora of potent soluble factors like cytokines, tumor necrotic factors, RONS, proteases like MMPs, leading to neoplastic progression (Munn, 2017) (Figure 1). Tumor-associated macrophages (TAMs), a major component of the tumor microenvironment, can generate a variety of angiogenic growth factors and proteases (Nowak and Klink, 2020). TAMs facilitate the progression of ovarian cancer at many stages of the disease development, including immune evasion of tumor cells and invasion of cancer cells. TAMs induce the invasive potential of ovarian cancer cells by secreting IL-6 and TGF-β, along with the production of MMPs (Nowak and Klink, 2020). TAMs upregulate MMP-2, -9, and -10 productions, through activating NF-κB, MAPK, and TLR signaling pathways in ovarian cancer (Ke et al., 2016).

The major inflammatory route implicated in gynecological carcinogenesis is the activation of the Cyclooxygenase-2 (COX-2)-Prostaglandin E2 (PGE2) pathway. Among the two COX isoforms, COX-1 helps in tissue integrity, proper platelet aggregation, and renal function, whereas COX-2 is predominantly expressed in inflammatory conditions and contributes to cancer development and metastasis. Upregulation of COX-2/PGE2 has been identified in many human cancers, including gynecological cancers (Liu et al., 2015; Ye et al., 2020). In early-stage uterine cervical cancer, greater levels of COX-2 expression are associated with a higher incidence of invasion and lymph node metastases (Parida and Mandal, 2014). For the cervical cancer cells to metastasize the malignant cells must have the capacity to move and infiltrate, disrupt intercellular connections, lyse extracellular matrix (ECM), and promote migration of endothelial cells and capillary lumen formation. MMP-9, and -2, and urokinase plasminogen activators play a crucial role in degrading the ECM and enabling metastatic cells to enter the vasculature and move into the target organ, resulting in tumor metastasis of cervical malignancies (Ye et al., 2020). Pentraxin 3 (PTX3), also known as the TNF-α inducible gene, is an inflammatory molecule involved in the immunological response, inflammation, and cancer. PTX3 has been known to have an essential role in tumor-associated inflammation. Positive expression of MMP-9 is linked with PTX3 expression in lung adenocarcinoma, suggesting an association of PTX3 with tumor grade and severity of malignancies (Ying et al., 2016). In human cervical cancer cells, knockdown of PTX3 reduced the tumorigenic and metastatic potential by downregulating MMP-2 and -9 activities. Furthermore, PTX-3 knockdown in mice showed reduced tumorigenicity and lung metastasis, indicating a crucial oncogenic role of PTX-3 (Ying et al., 2016).

G-coupled protein receptors (GCPR) associated with chemokine signaling are implicated in cancer migration, invasion, and metastasis (Bar-Shavit et al., 2016). Several inflammatory chemokines CCL2, CCL5, CXCL1, and CXCL12 have been implicated in gynecological cancer progression. In ovarian cancer cells, CXCL12 and its receptor CXCR4 increased the production of integrin-1β and vascular endothelial growth factor-C (VEGF-C), activating tumor vasculature in ovarian cancer (Predescu et al., 2019). In another report, CXCL5/CXCR2 axis promotes proliferation and migration of uterine cervix cells by regulating ERK and AKT pathways, contributing to its oncogenic potential (Feng et al., 2018). TAMs induce cancer cell migration by upregulating CCL20 production, activating CCR6 reception, and promoting EMT in ovarian cancer (Liu et al., 2020b). Upregulation of CXCR7 led to enhanced MMP-9 expression and cell adhesion and invasion in ovarian cancer (Yu et al., 2014).

Rapid tumor growth produces a tissue environment with low oxygen concentrations. Tumor hypoxia is known to have a role in tumor inflammation by regulating inflammatory mediator signals in both cancer and adjacent cells in the microenvironment (Jing et al., 2019). Under normoxic conditions, hydroxylation of the proline residues within the prolyl-hydroxylases of the HIF-1α takes place. This alteration enables the von Hippel-Lindau (VHL) to ubiquitinate suppressor protein that directs the proteasomal degradation of HIF-1/-2 subunits. In the absence of oxygen, HIF subunits stabilize and form heterodimers of HIF-1 and/or HIF-2 may then bind to hypoxia response element (HRE) of the target genes including ECM degrading enzymes, oncogenic growth factors, and macrophage-recruiting growth factors (DiGiacomo and Gilkes, 2018). MMP-13 is upregulated in ovarian cancer by HIF-1and leads to increased invasion and migration *in vitro* (Zhang et al., 2019).

### Therapeutic Strategies for Gynecological Cancer Treatment

In the early days, ovarian cancer treatment was mostly based on observations and opportunities, and there hasn’t been a randomized study comparing the effectiveness of debulking surgery with no surgery in advanced ovarian cancer. To this day, there is no effective therapy for HPV persistence. Cervical cancer prevention is based on the use of expensive HPV vaccinations and frequent cervical screenings, posing an economic burden on women in developing countries. (Hu and Ma, 2018). Development of precision medicine based on novel variations of conventional medicines and innovative therapeutic methods, including anti-angiogenic medicines, poly-ADP-ribose polymerase (PARP) inhibitors, growth factor signaling inhibitors, or folate receptor inhibitors, as well as numerous immunotherapeutic methods, are being trialed for use (Cortez et al., 2018).

The study of cancer risk among long-term users of non-steroidal anti-inflammatory drugs (NSAIDs) provides the strongest evidence supporting the importance of inflammation during neoplastic development. Overexpression of COX-2 and PGE2 has been seen in numerous human malignancies and pre-cancerous lesions, and COX inhibitory medications have been shown to protect against colorectal cancer and breast cancer (Eberhart et al., 1994; Sheng et al., 1997; Dirix et al., 2008). NSAIDs and selective COX-2 inhibitors delay the development of endometrial cancer, ovarian cancer, and cervical cancer (Daikoku et al., 2005; Hasegawa et al., 2005; Kim et al., 2013). Combined treatment of celecoxib (a COX-2 inhibitor) and rapamycin (a mammalian target of rapamycin complex one inhibitor, a mTORC1 inhibitor) reduces endometrial cancer (EC) progression in mouse models of EC and human EC cell lines (Daikoku et al., 2014) (Figure 1). However, in ovarian cancer, SC-560 (a COX-1 inhibitor) can suppress the production of PGE2 whereas while NS-398 and rofecoxib (COX-2 inhibitors) cannot, which suggests the COX-1 is the primary enzyme for producing PGE2 instead of COX-2 in ovarian cancer cells (Kino et al., 2005).

TNF- α is a major cytokine constitutively expressed in most malignant ovarian carcinomas and is responsible for cell proliferation, invasion, stemness by secreting cytokines, angiogenic factors, and MMPs (Madhusudan et al., 2005). Etanercept, a recombinant TNF receptor that binds to TNF- α and inactivates it, has shown therapeutic efficacy in recurrent ovarian cancer patients (Madhusudan et al., 2005). Since TNF- α exerts its effects primarily *via* modulating the NF-κB pathway, selective inhibitors of the NF-κB pathway, e.g., Bortezomib, have been tested in prostate cancer, myeloma, and ovarian cancer (Richardson et al., 2003; Bruning et al., 2009) (Figure 1). NF-κB/IκB signaling is an essential step for cancer cell survival and has been implicated in ovarian cancer development (De Simone et al., 2015; Tian et al., 2019). Resistance of cancer cells to chemotherapeutic agents has been associated with deregulated NF-κB activation (Zerbini et al., 2011).

IL-6 is produced in response to local proinflammatory cytokines such as TNF-α, within the tumor microenvironment and can activate JAK/STAT3, MAPK, and PI3K/AKT pathways (Johnson et al., 2018). Siltuximab, the monoclonal antibody with a high binding affinity for IL-6, showed therapeutic activity in patients with platinum-resistant ovarian cancer in Phase II clinical trial (Nowak and Klink, 2020) (Figure 1). IL-6 induced activation of JAK/STAT3 pathway *via* IL-6R dimerization leads to JAK2 recruitment, followed by activation and translocation of STAT3 into the nucleus, where it alters expression of genes involved in proliferation, migration, differentiation, angiogenesis, stemness, and chemoresistance (Jin, 2020; Sreenivasan et al., 2020). Targeted gene therapy using a cationic solid lipid nanoparticle system encapsulating STAT3 decoy oligonucleotides has shown to inhibit cell invasion by modulating E-cadherin and SNAI1 transcription factors and MMP-9 expression, suppressing growth and causing cell death through apoptosis and autophagy (Ma et al., 2015).

Over the years, several MMP inhibitors (MMPIs) have been discovered and trialed for use as therapeutic strategies in various cancers. Batimastat, one of the first MMPIs synthesized, had a broad range of inhibition against most MMPs and early clinical trials showed a low oral availability. Marimastat, a next-generation drug, was taken for phase 2 and 3 trials for metastatic solid tumors in lung, breast, brain, colorectal cancers. Other more specific MMPIs, such as tanomastat, a small molecule inhibitor of MMP-2, -3, -8, -9, and -13, prinomastat, which inhibits MMP-2, -3, -9, -13, and -14, and rebimastat, an inhibitor of MMP-1, -2, -3, -8, -9, -13, and -14, were then tested, in cancers (Winer et al., 2018). Ovarian carcinoma patients with platinum/paclitaxel chemotherapy were treated with BAY 12-9566 (tanomastat), showed no impact on overall survival (Hirte et al., 2006). Inhibition of non-specific targets of MMPIs, such as ADAMs and other MMPs, was the main reason behind severe side-effects, e.g., musculoskeletal syndrome, of these drugs. The advent of a new generation of MMPIs preferentially targets MMP ‘exosites’ rather than the conserved catalytic area, thereby blocking the unique function of a single MMP, showed promise in several diseases (Levin et al., 2017). In the mouse models of colorectal cancer, humanized selective monoclonal antibodies against MMP-9 (AB0041, GS-5745) were found to be effective. DX-2400 is a high-affinity, highly specific inhibitor of MMP-14 that inhibits tumor development and metastasis *in vivo* animal models of breast cancer and melanoma (Winer et al., 2018). D1 (A12), a therapeutic monoclonal anti-ADAM17 antibody, was tested in a mice model of ovarian showed inhibition of tumor growth (Richards et al., 2012). Computational approaches to design very specific, small-molecule inhibitors and testing their efficacy in gynecological malignancies are also being conducted *via* high-throughput screens (Bursavich and Rich, 2002). Together these drugs show promise for future use by preventing side effects and by minimizing inhibition of protective MMPs. In future, personalized anticancer therapies targeting individual metalloproteinases upregulated in tumors should be adopted for a better success of such inhibitors.

### Effects of Metalloproteinases and ADAMs in Inflammation and Cancer

Metalloproteinases play a crucial role in ECM remodeling in both the normal physiological condition as well as in pathological conditions including various gynecological disorders like polycystic ovary syndrome (PCOS), preeclampsia, spontaneous abortions, and cancer. An increase in MMP-2 and -9 is seen during normal pregnancy to support vasodilation, placentation, and uterine expansion. However, a higher MMP-9 expression level can cause spontaneous abortions. The expression of MMPs gets altered during the complications in pregnancy and a decreased MMP-2 and -9 leads to vasoconstriction, hypertensive pregnancy, and preeclampsia (Chen and Khalil, 2017; Balci and Ozdemir, 2019). MMPs and their inhibitors are said to be involved in the pathogenesis and follicular development of PCOS. A higher MMP-9/TIMP-1 ratio and a decreased ADAMTS-1 have been found in PCOS patients therefore might be involved in their pathogenesis (Xiao et al., 2014; Ranjbaran et al., 2016). Metalloproteinases are key regulators for many of the changes in the tumor microenvironment, inflammation, and metastasis. The major source of MMPs, ADAMs, as well as TIMPs, is from the stromal cells that infiltrated the tissue, although cancerous cells can also express them (Egeblad and Werb, 2002). Depending on the tumor types different stromal cells secrete different metalloproteinases and their inhibitors into the extracellular matrix for the specific alteration of the milieu around the tumor. These secreted metalloproteinases show some major consequences on their activity and function; for example, neutrophil-derived MMP-9 easily gets activated due to the absence of a bound TIMP-1 molecule (Ardi et al., 2007). Metalloproteinases are regulated at different stages such as at their gene expression level, their localization, while converting from their pro-active zymogen to its active form, or by the expression of their inhibitors; and the specific context is needed while studying their pathophysiological relevance. Few of the MMPs such as MMP-2, -3, -7, -9, and -12 might provide a negative feedback loop as well. The complexity of the MMP regulation is important considering that the activity of the MMPs that are secreted from the inflammatory cells can damage the tissue and prolong the inflammatory response in chronic inflammatory diseases and cancer (Parks et al., 2004). This inflammatory response leads to the production of a large amount of ROS by the macrophages and neutrophils at the tumor site which further influences the function of MMPs by activating them *via* oxidation of the pro-domain cysteine and amino acid modification at its catalytic domain (Kessenbrock et al., 2010). Studies showed a prevalence of M1 cells subtype among the tumor-associated macrophages in epithelial serous ovarian cancer microenvironment and are also associated with increased IL-6 levels in patients with advanced stages. In the pre-clinical studies of OC models, it is seen that the M1 macrophages increased the metastatic potential of the cancerous cells by activating NF-κB whereas M2 subtypes were related to the cancer cell progression and the formation of spheroids. This change of subtype polarization in OC patients with advanced stages might be helpful in the prognosis of the disease and are likely to respond to chemotherapy (Maccio et al., 2020). The upregulation in the MMP secretion following the release of the inflammatory mediators such as chemokines or cytokines supports the presence of a feedback loop for tightly controlling the activities of the chemokines by the MMPs which further influence the immune response. This chemokine processing at N-/or C-terminus by the MMPs shows different effects on chemokine activity, the changes in the chemokine gradient, and the recruitment of the inflammatory cells in the inflammatory tissue, and can also regulate inflammation, and leukocytes transmigration from vasculature to tissue (Butler and Overall, 2013; Nissinen and Kahari, 2014).

Several MMPs play a role in both the pro-and anti-inflammatory pathways. For example, MMP-8 regulates skin inflammation whereas MMP-9 is shown to have an anti-inflammatory role in the inflammation of skin and glomerulonephritis (Nissinen and Kahari, 2014; Kapoor et al., 2016). Inflammation and cancer are interlaced with each other therefore when tumorigenicity and/or metastasis are increased by the inflammatory components in the tumor microenvironment, the MMPs also show their anti-inflammatory role to potentially decrease the tumorigenicity and metastasis interfering with the progression of cancer. For example, MMP-3 deficient animals with squamous cell carcinoma showed increased proliferation, tumor growth, and metastasis. Again, an overexpression of MMP-26 increased the survivability of the patient by the proteolytic degradation of ER-β in ductal carcinoma *in situ*. MMP-2 and -12 act on plasminogen to produce angiostatin, an anti-angiogenic component, which suppresses metastasis and tumor growth in lung cancer (Dufour and Overall, 2013).

There is an increase in stiffness of the tissue and interstitial fluid pressure, and an alteration in the blood flow is also seen with increased tumorigenicity in addition to the mechanical forces that further contribute to the progression of the tumor *via* proteolytic degradation of the ECM thereby altering the conformation of the substrates of the MMPs and empowering the easy recognition and proteolytic cleavage of the substrate proteins (Butcher et al., 2009). Von Willebrand factor (VWF) is a multimolecular complex, crucial for the regulation of blood clotting mechanism, is highly sensitive to the increased shear forces in the blood flow which is usually seen during an injury. The high shear forces of the blood unfold the VWF domain two by inducing the conformational changes of the complex which leads to an exposed cleavage site for ADAMTS-13. ADAMTS-13 then starts cleaving the complex into monomers thereby initiating the clotting of the blood (Zhang et al., 2009). Furthermore, the ADAM and ADAMTS family members are also correlated with the progression of the tumor. Therefore, the involvement of the mechanical forces in the regulation of the activity and function of the MMP is plausible in inflammation and cancer metastasis (Kessenbrock et al., 2010).

**FIGURE 2:**
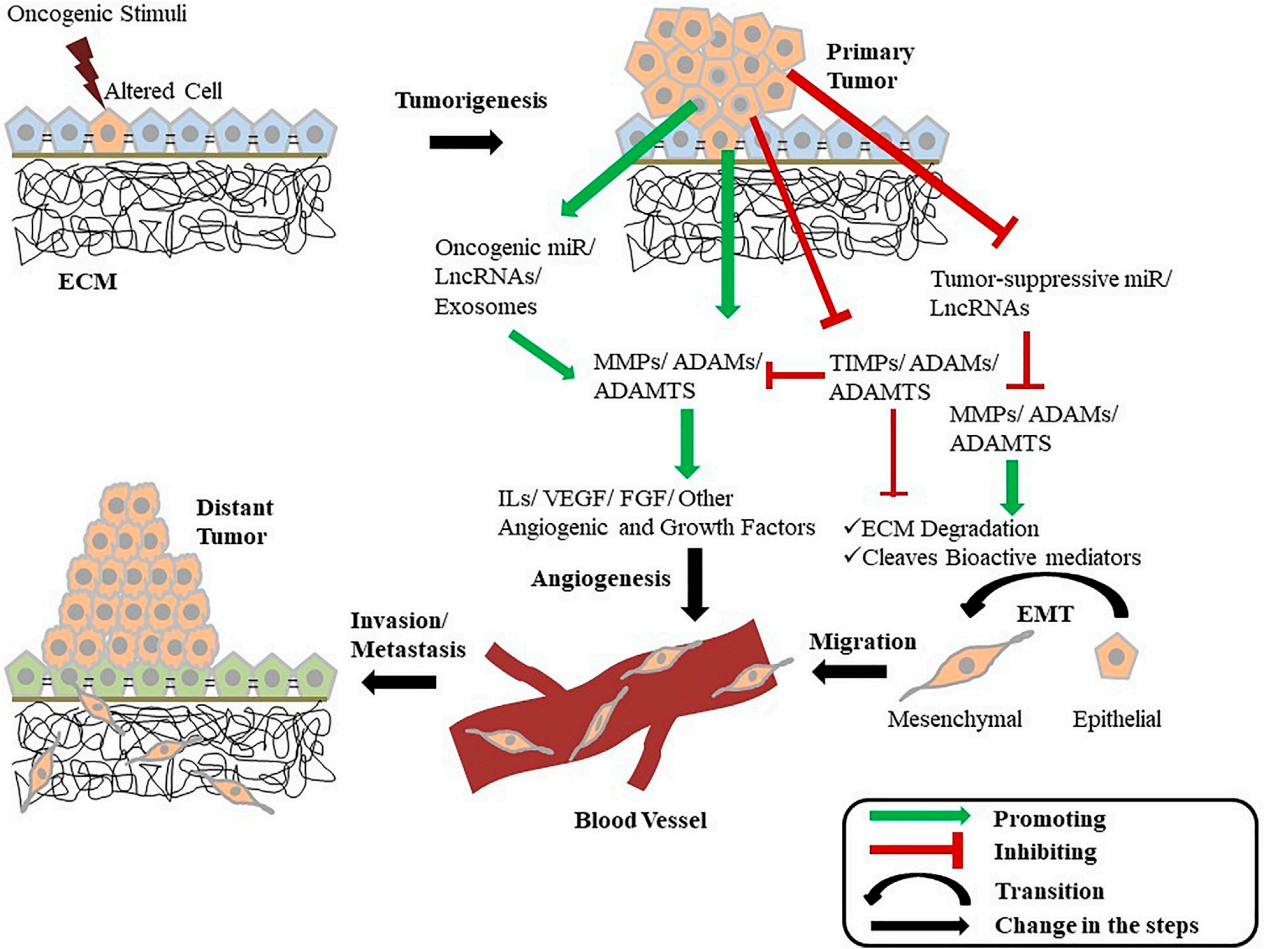
Diagrammatic illustration showing the intertwined relationship of metalloproteinases with cancer. A normal cell gets altered and becomes tumorigenic when gets exposed to an oncogenic stimulus. These tumorous cells can now enhance the activity of multiple growth-promoting MMPs, ADAMs, and ADAMTS while inhibiting the function of their inhibitors (TIMPs) and growth-inhibiting metalloproteinases. The tumor cells also promote the expression of certain miRNAs and LncRNAs that increases the expression of MMPs, ADAMs, and ADAMTS as well as inhibit the expression of tumor-suppressing miRNAs and LncRNAs. These cells also release exosomes containing various MMPs and ADAMs. The secreted metalloproteinases promote ECM degradation and also cleave various other bioactive mediators promoting EMT and tumor progression. Metalloproteinases further influence the release of various cytokines, angiogenic factors, and other growth factors, leading to the activation of angiogenesis and other cellular pathways associated with tumor growth. This EMT and angiogenesis mediated by the MMPs and ADAMs provide the cell the ability to invade and metastasize in distant tissues. ECM, Extra-Cellular matrix; MMP, Matrix metalloproteinase; ADAM, A Disintegrin and metalloproteinase; ADAMTS, A Disintegrin and metalloproteinase with Thrombospondin Motif; TIMP, Tissue Inhibitors of metalloproteinase; miR, MicroRNA; LncRNA, Long Non-Coding RNA; IL, Interleukin; VEGF, Vascular Endothelial Growth Factor; FGF, Fibroblast Growth Factor; EMT, Epithelial-to-Mesenchymal Transition.

The complexity of the function of MMPs is discovered when it is seen to be doing more than only degrading the physical barriers. Metalloproteinases have a key role in multiple cellular pathways. Metalloproteinases are critically involved in the misbalance between the growth and anti-growth signals, the metastatic spread, apoptosis, and angiogenesis in cancerous tissues (Kessenbrock et al., 2010; Kapoor et al., 2016; Winer et al., 2018). The upregulated tumor proliferation can be achieved by either becoming self-sufficient in growth-promoting factors or by acquiring insensitivity to the anti-growth factors (Winer et al., 2018) (Figure 2).

#### Tumorigenic Role of Metalloproteinases

Evidence suggests the major involvement of MMPs in the regulation of innate and acquired immunity. The progression of cancer is linked to the process of inflammation and cytokine production by immune cells in many ways. Studies in knockout mice support the key role played by MMPs in acute and chronic inflammation (Kessenbrock et al., 2010). The proteolytic cleavage and conversion of pro-inflammatory cytokine pro-TNF-α expressed as a membrane-bound precursor in T cells and/or macrophages, into its soluble cytokinetic form are mediated by ADAM-17, and specific MMPs (Manicone and McGuire, 2008). TNF-α is produced by tumor cells in abundance promoting cell survival in an NF-κB-dependent mechanism suggesting the crucial role of ADAM-17 and MMPs in promoting tumorigenesis (Kessenbrock et al., 2010).

#### Metastatic Role of Metalloproteinases

Tumor cells when gains invasiveness enter the bloodstream where they can easily invade through the vascular basal lamina thereby can spread into the distant tissues and promoting metastasis by deregulating the metalloproteinases which can now also spread beyond their microenvironment. MMP-2, -9, and -14 degrade the vascular basal lamina. Several MMPs including -9, -10, and -15 are involved in the degradation or decrease in the synthesis of E-cadherin levels in various tumors which further promotes metastasis (Winer et al., 2018). MMPs can potently influence multiple fundamental signaling pathways such as mitogen-activated protein kinase (MAPK) signaling, epidermal growth factor receptor (EGFR) pathway, transforming growth factor-β (TGF-β) pathway, and nuclear factor-KB (NF-κB) signaling activation during immune responses activation (Kapoor et al., 2016). TGF-β pathway plays a key role in maintaining the tissue homeostasis by exerting a tumor-suppressive function in normal tissue whereas in tumor malignancy the genome of the TGF-β receptor often gets mutated leading to the unresponsiveness of the receptor to TGF-β. Furthermore, TGF-β can also act as a tumor-promoting factor in non-malignant stromal cells by evading the immune surveillance and thereby gets exploited by the tumor leading to increased metastasis and invasion (Kessenbrock et al., 2010). The EGFR ligands also act as important regulators of tissue homeostasis and the genetic mutation in the molecules involved in this pathway is often seen in breast cancer and some other malignant diseases (Hynes and Lane, 2005). ADAM proteinases are seen to play a key role in regulating the EGFR pathway, for instance, ADAM-10 induces the soluble EGF release while ADAM-17 converts other EGFR ligands including TGF-α and epiregulin from its pro-forms to its active form (Sahin et al., 2004). In ovarian cancer, activated EGFR upregulates MMP-9 which sequentially promotes the degradation of E-cadherin molecules resulting in metastasis (Cowden Dahl et al., 2008; Alshenawy, 2010).

#### Angiogenic Role of Metalloproteinases

MMPs not only degrades the ECM, which allows the detachment and migration of the cells, but also contributes to angiogenesis by promoting the release of pro-angiogenic factors that are bound to the ECM such as basic fibroblast growth factor (bFGF), vascular endothelial growth factor (VEGF), and TGF (Kapoor et al., 2016). Angiogenesis, the formation of new capillary blood vessels from the already existing vessels; requires multiple interactions between endothelial cells, stoma and vascular cells, surrounding pericytes, smooth muscle cells, ECM, and angiogenic growth factors (Kapoor et al., 2016; Winer et al., 2018). The major MMPs involved in the angiogenic pathway are MMP-2, -9, and -14, and to some extent MMP-1 and -7 (Kessenbrock et al., 2010; Winer et al., 2018). MMP-2 upregulates vascular endothelial growth factor-A (VEGF-A) secretion by the tumor cells in human melanoma leading to the interaction of the melanoma cells with the lining of the blood vessels and favoring their extravasation (Lee et al., 2011; Desch et al., 2012). MMP-9 induces angiogenesis by mediating the bioavailability of VEGF for its receptor VEGFR2. Apart from its role in angiogenesis MMP-9 is also involved in vasculogenesis making it an important therapeutic target for cancers. On the contrary, MMP-12 produces angiostatin, an anti-angiogenic factor, showing its suppressive function on lung metastasis. Therefore metalloproteinases may possess both the angiogenesis-promoting and–inhibiting role depending on the MMP expression time frame and its substrate availability (Kessenbrock et al., 2010). The pro-angiogenic factors VEGF, FGF-2, and TGF-β released from the ECM are isolated in the stroma where these factors get mobilized by the MMPs which creates a favorable metastatic niche supporting the growth of the tumorous cells (Winer et al., 2018).

#### Anti-Apoptotic Role of Metalloproteinases

MMP-7 cleaves Fas ligand and removes it from the cell surface, which therefore cannot stimulate the Fas death receptor, involved in the activation of innate apoptosis, leading to the survival of tumor cells. The malignant cell evades apoptosis and might acquire some chemotherapeutic resistance with the help of this mechanism in non-small cell lung cancer patients (Kessenbrock et al., 2010; Winer et al., 2018). MMP-14 also shows the anti-apoptotic function and thereby promotes tumor growth (Winer et al., 2018). ADAM-10 is also involved in Fas ligand degradation and therefore might also repress the induction of apoptosis by the cytotoxic lymphocytes (Kessenbrock et al., 2010).

### Involvement of Matrix Metalloproteinase in Gynecological Cancer

The ECM is a complex compartment with multiple functions involved in various biochemical pathways both in the normal cellular condition as well as in the tumor microenvironment. ECM remodeling can greatly affect tumor growth, invasion, migration, metastasis, angiogenesis, and apoptosis *via* various cellular processes thereby promoting cancer progression. For example, Cancer-associated fibroblasts (CAFs) can enhance MMP expression as well as its activation which in turn cleaves type-I collagen, an ECM component, resulting in ECM degradation and remodeling allowing cancer cells to invade and migrate to a distant site (Erdogan and Webb, 2017; Di Martino et al., 2021). MMP-2, -9, and -14 TIMP-1 and -2 have been seen to be altered in cell proliferation, invasion, and metastasis in multiple types of cancers.

#### Cervical Cancer

In cervical cancer (CC) an upregulated MMP-2 and TIMP-2 levels in the stroma are related to a decrease in patient survivability. High MMP-9 expression levels can also be used as a diagnostic factor in CC patients (Azevedo Martins et al., 2020). MMP-2 induces ECM degradation and activates tissue damage mechanisms by regulating multiple interleukin releases including IL-8 leading to invasion and metastasis. A high expression of MMP-2 and lower expression of circulating IL-8 is seen in HPV-associated CC patients (Shukla et al., 2020). The HPV-16 oncoproteins E6 and E7 were also found to downregulate TIMP-2 and RECK (reversion-inducing cysteine-rich protein with kazal motifs) levels, a membrane-bound protein that inhibits MMP function, and upregulate MMP-9 activity in HPV-associated CC lesions (Cardeal et al., 2012). In the early stages of CC, metastasis of lymph nodes (LN) is seen to be associated with poor prediction. RNA sequencing of LN negative and LN-positive patient samples showed an aberrant expression of multiple genes among which MMP-1 is constantly overexpressed along with the peroxisome proliferator-activated receptor (PPAR) signaling pathway which is found to be associated with lymph node metastasis in CC patients. Studies revealed that MMP-1 knockdown results with an increased E-cadherin and decreased vimentin expression in cervical cancer cell lines which leads to a decrease in cell proliferation, migration, and invasion which suggests that MMP-1 plays a crucial role in regulating epithelial-to-mesenchymal transition (EMT) (Tian et al., 2018). Upregulation in the MMP-7 and -9 expressions are significantly correlated with LN metastasis in association with the higher expression of Ki67, which induces cellular proliferation, promoting tumor invasiveness in early CC cases (Guo et al., 2018). Aberrant expression of micro-RNAs has also been found in multiple cancer types and is often associated with the regulation of MMPs. In the serum and tissues of metastatic CC patients and CC cell lines miR-G-10 is found to be highly overexpressed which significantly promotes migration, invasion, and anoikis resistance *in vitro* and lung metastasis *in vivo*. miR-G-10 binds to the 3′ UTR of PIK3R3 and activates AKT/NF-κB signaling pathway in a GRSF1-dependent manner *via* upregulating PI3KR3. This miR-G-10 suppresses TIMP-3 in the AGO-2 complex and thereby regulates MMP-9 expression in CC (Sun et al., 2019). The chemokine receptor CXCR4 and its only ligand stromal cell-derived factor-1 (SDF-1)-alpha plays some crucial role in inflammation, hematopoiesis, infection of HIV, invasion, migration, and metastasis in some malignancies including CC. CXCR4/SDF-1alpha regulates the adhesion capability of the cells by activating the MAPK/ERK signaling pathway which in turn promotes MMP-2 secretion thereby inducing invasion and metastasis in CC (Shen et al., 2008).

#### Endometrial Cancer

VEGF and MMPs are the major angiogenic factors and play a key role in angiogenesis especially during endometrial degeneration and remodeling and during and after the menstrual cycle. However, in cancer hypoxia-induced factors (HIF) activate the “angiogenic switch,” since hypoxia promotes tumor, by stimulating VEGF and MMPs leading to the proliferation and migration of vascular endothelial cells and inducing more blood vessel formation to supply the nutrients and oxygen to the tumor cells. In endometrial cancer (EC) VEGF and MMPs play a major role in tumor growth and metastasis since MMP-2 and -9 are persistently upregulated in EC although the expression levels are closely associated with the clinical stage, tumor invasion, and metastasis of the disease (Mahecha and Wang, 2017; Liu et al., 2018).

#### Ovarian Cancer

Ovarian Cancer (OC) has a high mortality rate due to poor prognosis and aberrant MMP expression is seen to enhance tumor growth, invasion, metastasis, and malignancy (Al-Alem and Curry, 2015). Researchers have found an imbalance between MMPs and TIMPs in malignant tissue (Zhang and Chen, 2017). In OC, HIF-1α activates its downstream MMP-13 promoting invasion and metastasis under the hypoxic condition which makes it a favorable target for effective therapeutic strategy (Zhang et al., 2019). Epithelial ovarian cancer (EOC) cells directly come across the human peritoneal mesothelial cells (HPMC) in the peritoneal cavity to initiate the metastatic process. OC cells-derived exosomes morphologically change HPMCs, upon entering the cells, into a mesenchymal, spindle phenotype. These exosomes are enriched with CD44 which induces HPMCs to secrete MMP-9 and thereby promotes invasion (Nakamura et al., 2017) (Figure 2). Significant levels of serum cathepsin L (CL), heparanase (Hpa), and MMP-9 are found in EOC malignancies which are associated with peritoneal as well as distant metastasis. These elevated serum levels of CL, Hpa, and MMP-9 before surgery may act as potential markers to determine the extent of extra-pelvic metastasis in OC (Zhang et al., 2011). Advanced OC is highly metastatic due to multiple reasons one of them being the release of cancer-derived extracellular vesicles (EVs) from the highly metastatic cells which acts as a crucial metastatic mediator in moderately metastatic tumor cells. Notably, these EVs are packaged with abundant MMP-1 mRNAs and the MMP-1 expression is highly correlated with a poor prognosis of OC (Yokoi et al., 2017). Additionally, a low expression level of miR-508-3p is also seen in OC tissues along with a high expression of cyclin A2 (*CCNA2*) and MMP-7 levels. Although CCNA2 and miR-508-3p are said to be the independent predictors in OC patients, miR-508-3p directly binds to the 3′-untranslated region (UTR) of CCNA2 to suppress the cellular proliferation while it directly targets the 3′-UTR of MMP-7 to suppress the migration and invasion of the tumor cells suggesting a tumor-suppressing role of miR-508-3p in OC (Guo F. et al., 2020). Recent studies have shown the diverse role of long noncoding RNAs (lncRNAs) in regulating the development of cancer. On one hand, upregulation of lncRNA P73 antisense RNA 1T (TP73-AS1) is observed in tumor tissue of OC patients and is related to increased cellular proliferation, migration, and invasion, and thereby poor prognosis of the disease. It has been evidenced that overexpression of TP73-AS1 significantly upregulates MMP-2 and MMP-9 which in turn promotes invasion and migration in OC tissues (Wang et al., 2018). Contrary to TP73-AS1, a lncRNA named AOC4P is downregulated in epithelial ovarian cancer (EOC) tissues which are positively correlated to the LN metastasis and advancement in the disease stage *via* activated EMT (Figure 2). This EMT is executed by the altered MMP-9 and COL-1A2 expression tightly regulated by AOC4P which proves the anti-metastatic effect of AOC4P in EOC (Lin et al., 2020). CA125 is a widely used biomarker among OC patients; however, it is not always seen to be elevated in all OC patients. On the other hand, the urinary MMP-2 and -9 are found in very high levels in OC patients with normal CA-125 levels. Hence, it is clinically useful for the prediction of advanced or recurrent OC (Coticchia et al., 2011).

### Involvement of ADAMs in Gynecological Cancer

ADAM-8 is seen to be overexpressed in the inflammatory cell which can be used as a marker for the prediction of intra-amniotic inflammation during labor (Malamitsi-Puchner et al., 2006). An overexpression of ADAMTS-1, a member of the ADAMTS gene family of metalloproteinases, can also be seen in the cervix during labor suggesting the multiple functions of extracellular matrix proteins in modulating integrity of the tissue, differentiation, and migration of the epithelial cells and inflammation (Ruscheinsky et al., 2008). ADAMTS-1 regulates cytokine-mediated decidual ECM remodeling. Interleukin-1β (IL-1β), a pro-inflammatory cytokine, induces ECM degradation whereas the anti-inflammatory cytokine TGF-β1 counterbalanced the effect of IL-1β thereby regulating the expression of ADAMTS-1 (Ng et al., 2006).

#### Cervical Cancer

ADAMs play a central role in ECM degradation and metastasis. A consistently positive expression of ADAM-9 has been found in malignant CC, cervical intra-epithelial carcinomas, and squamous cell carcinomas and therefore can be used as a prognostic factor. Whereas miR-126 specifically targets ADAM-9 gene thereby can negatively regulate CC cell proliferation (Mohd Isa et al., 2019; Guo T. et al., 2020). ADAM-17 stimulates the activity of EMMPRIN, AREG, p-EGFR, p-ERK, MMP-2, and MMP-9 proteins contributing to the progression of the disease. Studies revealed an upregulated ADAM-17, amphiregulin (AREG), extracellular matrix metalloproteinase inducer (EMMPRIN), and MMP-9 expression markedly correlated with the advanced stages, invasion, LN metastasis, and poor survivability in patients with uterine cervical carcinoma (Xu et al., 2014).

#### Endometrial Cancer

The overexpression of ADAM-19 is found in patients with endometrial cancer which is possibly correlated with the progression and diagnosis of endometrial carcinoma therefore might be used as a promising marker for the prognosis of the disease (Ou et al., 2008). ADAMTS-9 was found to be significantly decreased in patients with endometrial polyps (EPs), a benign gynecological disorder. Moreover, ADAMTS-9 functions as a tumor-suppressing gene in multiple malignancies by regulating ECM degradation, vascular biology, and inflammation. Furthermore, a reduced expression of ADAMTS-9 protein induces the pathogenesis of Eps (Tokmak et al., 2019). Endometriosis, also a benign gynecological disease, is correlated with aberrant expression of mRNA and long non-coding RNA (lncRNA). Among multiple lncRNAs and mRNAs that are found to be dysregulated in endometriosis, ADAMTS-7, tumor protein p53 (Tp53), distal-less homeobox 3 (Dlx3), and pyrimidinergic receptor P2Y6 (P2ry6) proteins, which are co-expressed, were said to be associated with endometriosis-related inflammation and reproductive pathways (Cai et al., 2019).

#### Ovarian Cancer

ADAM-10 and 17 are known to cleave the cell adhesion molecule Nectin-4, which is overexpressed on OC tumors and releases the soluble Nectin-4 (sN4). This cleaved domain is detected in the serum of OC patients. Nectin-4 plays a key role in the lysophosphatidic acid (LPA) mediated AKT signaling pathway stimulation which subsequently promotes EMT. Both the ADAMs also stimulate the EGFR ligands further activating the AKT and ERK signaling pathways. Studies suggest that phosphorylation of EGFR, AKT, and ERK can be reduced by targeting Nectin-4 in OC which makes Nectin-4 a promising target for therapeutic strategy (Buchanan et al., 2017). Additionally, ADAM-10 and -17 have a role in the regulation of CxCL-16 and its receptor CXCR6. A high serum sCXCL-16 (soluble form) is found in patients with metastatic OC. In OC malignant cells ADAM-10 and -17 inhibit the shedding of sCXCL-16 promoting migration and metastasis making ADAM a favorable target for inhibition of migrating cells (Gooden et al., 2014). Reports suggest that ADAM-23 is also expressed in multiple tumor types whereas in epithelial ovarian cancer (EOC) a lower expression level of ADAM-23 is seen. This loss of ADAM-23 expression in correlation with the advancement of the disease stage and lymph metastasis is possibly associated with the progression of EOC and therefore might be used as a promising prognostic factor in the detection of EOC (Ma et al., 2018).

## Conclusion

Activities of the metalloproteinases and their association with different physiological and pathophysiological conditions have been reported including inflammation and cancer for a few decades. Metalloproteinases are found either in their latent inactive form, active form, and/or in complex with its inhibitors (such as TIMPs), thus regulation of which becomes aberrant during pathological conditions. Metalloproteinases are crucial for maintaining biological homeostases such as wound healing, morphogenesis, immune responses, and angiogenesis. Proteolytic cleavage of the transmembrane proteins is the key mechanism for the regulation of different biological functions. Interaction between cell-cell and cell-matrix is of supreme importance for understanding the pathogenesis of various complex diseases including gynecological cancer. Since metalloproteinase (MMPs, ADAMs, and ADAMTS) cleaves a variety of substrates, including ECM components, various cytokines, chemokines, and other important biological molecules (Figure 2). Hence, MMPs and ADAMs have gained much attention and appeared to be the chief regulatory hub in inflammation and cancer progression as well as metastasis.

The recruitment of immune cells at the site of inflammation is the major characterization of inflammatory responses. The mechanism requires complex cell-cell and cell-substratum interactions for the recirculation and endothelial and epithelial transmigration of leukocytes. The migration of these immune cells is a complex process that involves ECM remodeling by matrix-degrading enzymes, various cytokines, and chemokines which underlie the key mechanism of inflammation (Charrier-Hisamuddin et al., 2008). In addition, chronic inflammation is associated with multiple cancer types including gynecological cancers. As metalloproteinases play a pivotal role in cancer progression and metastasis, we address the expression profiles of MMPs and ADAMs/ADAMTS in inflammation and gynecological cancer.

As evidenced, MMPs and ADAMs are found to be aberrantly expressed in the tumor microenvironment and promote migration, invasion, and metastasis in different gynecological cancers (Table.1). The process of angiogenesis is also closely correlated with cancer invasion and metastasis and is tightly regulated by MMPs and ADAMs (Figure 2). Therefore, identifying the specific mediators of cell migration, invasion and metastasis are of utmost importance to understand the underlying mechanism of inflammation and cancer development and determine the therapeutic strategies. This review highlights the role of MMPs and ADAMs/ADAMTS in inflammation and progression of different gynecological malignancies in terms of ECM degradation and secretion of various biological mediators involved in multiple cellular pathways. Aberrant expression of specific MMPs and specific ADAMs can be used as prognostic markers which makes them an attractive therapeutic target for the development of inhibitors. However, metalloproteinases have a diverse role in pathophysiological conditions. It can act as a pro-inflammatory or anti-inflammatory in different cancer types therefore simply inhibiting the metalloproteinases might be detrimental at the same time and a precise conceptualization is needed to understand the specific functions of metalloproteinases in specific cancer types before designing the clinical trial protocols of their inhibitors (Fields, 2019). Another indirect way to inhibit metalloproteinase expression is to target the pro-inflammatory mediators, such as cytokines or growth factors responsible for the upregulation of MMPs and ADAMs in metastasis, however, these factors also show diverse effects limiting the use of their inhibitors as therapeutics (Charrier-Hisamuddin et al., 2008). Therefore, appropriate therapeutic strategies require a detailed understanding of the function of MMPs and ADAMs in gynecological cancer progression which can help to develop inhibitors that bind specifically to certain metalloproteinases without cross-reacting with other metalloproteinases. One plausible way to achieve that goal is to develop the inhibitors in such a way that they can bind to the specific binding sites of the metalloproteinases other than their active domains. It is also evidenced that the metalloproteinases are critically associated with numerous biological factors therefore the use of a combination of specific drugs along with the inhibitors would be more effective approach and can increase the therapeutic efficacy.

**TABLE 1:** Involvement of metalloproteinases in the regulation of gynecological malignancies.

MMPs/ADAMs	Disease	Role	Mode of action	References
MMP-2↑	Cervical cancer	Tumor-promoting	↓IL-8, ↑ERK, ↑SDF-1α	Azevedo Martins et al. (2020); Shukla et al. (2020); Shen et al. (2008)
	Endometrial cancer	Tumor-promoting	↑VEGF	Mahecha and Wang (2017); Liu et al. (2018)
	Ovarian cancer	Tumor-promoting	↑TP73-AS1	Wang et al. (2018)
MMP-9↑	Cervical cancer	Tumor-promoting	↓TIMP-2,-3, RECK,↑Ki67, ↑NFκB	Cardeal et al. (2012); Guo et al. (2018); Azevedo Martins et al. (2020); Sun et al. (2019)
	Endometrial cancer	Tumor-promoting	↑VEGF	Mahecha and Wang (2017); Liu et al. (2018)
	Ovarian cancer	Tumor-promoting	↑CD-44	Nakamura et al. (2017)
MMP-1↑	Cervical cancer	Tumor-promoting	↑Vimentin,↓ E-cadherin, ↑EMT	Tian et al. (2018)
	Ovarian cancer	Tumor-promoting	—	Yokoi et al. (2017)
MMP-7↑	Cervical cancer	Tumor-promoting	↑Ki67	Guo et al. (2018)
	Ovarian cancer	Tumor-promoting	↓mir-508-3p	Guo et al. (2020a)
MMP-13↑	Ovarian cancer	Tumor-promoting	↑HIF-1α	Zhang et al. (2019)
ADAM-9↑	Cervical cancer	Tumor-promoting	↓mir-126	Mohd Isa et al. (2019); Guo et al. (2020b)
ADAM-17↑	Cervical cancer	Tumor-promoting	↑EMMPRIN, AREG, pEGFR, pERK	Xu et al. (2014)
	Ovarian cancer	Tumor-promoting	↑SolubleNectin-4,↑AKT,ERK, ↑CXCL-16	Gooden et al. (2014); Buchanan et al. (2017)
ADAM-19↑	Endometrial cancer	Tumor-promoting	—	Ou et al. (2008)
ADAM-10↑	Ovarian cancer	Tumor-promoting	↑SolubleNectin-4,↑AKT,ERK, ↑CXCL-16	Gooden et al. (2014); Buchanan et al. (2017)
ADAM-23↓	Epithelial ovarian cancer	Tumor-suppressing	—	Ma et al. (2018)

## Abbreviations

MMP, matrix metalloproteinase; ADAMs, A disintegrin and metalloproteinase; ADAMTS, A disintegrin and metalloproteinase with thrombospondin motif; TAM, tumor-associated macrophage; ECM, extra-cellular matrix; CC, cervical cancer; OC, ovarian cancer; EOC, epithelial ovarian cancer; EMT, epithelial-to-mesenchymal transition; ILs, interleukins; NF-κB, nuclear factor kappa B; VEGF, vascular endothelial growth factor.
